# A Calorimetric Characterization of Cr(VI)-Reducing *Arthrobacter oxydans* at Different Phases of the Cell Growth Cycle

**DOI:** 10.1100/tsw.2003.33

**Published:** 2003-05-27

**Authors:** Nugzar G. Bakradze, Marina K. Abuladze, Victor M. Sokhadze, Nina V. Asatiani, Nelly A. Sapojnikova, Tamara M. Kartvelishvili, Emma N. Namchevadze, Nelly Y. Tsibakhashvili, Leila V. Tabatadze, Lia V. Lejava, Hoi-Ying Holman

**Affiliations:** ^1^ Department of Physics of Biological Systems, Andronikashvili Institute of Physics, AS of Georgia, 6 Tamarashvili st., Tbilisi, 380077, Georgia; ^2^ Institute of Molecular Biology and Biophysics, AS of Georgia, 14, Gotua st., Tbilisi, 380069, Georgia; ^3^ Lawrence Berkeley National Laboratory, University of California, Berkeley, USA

**Keywords:** bacteria melting, calorimetric study of bacteria, A. oxydans

## Abstract

This is the first of a series of calorimetric studies designed to characterize and understand survival mechanisms of metal-reducing bacteria isolated from metal-polluted environments. In this paper we introduce a new concept of thermal spectrum of the endothermic melting of *complex biological systems (e.g., proteins, nucleic acids, ribosomes, membrane structures)* in intact cells. All *thermal spectra measured are thermograms that describe the temperature dependence of heat capacity change of the complex systems of biologically active substances in bacterial cells*. This new concept of thermal spectrum was applied to investigate spectral features from intact cells of Cr(VI)-reducer Arthrobacter oxydans at different points of their growth conditions and stages. Over the temperature range of 40–105^°^C, we observed that spectral changes are particularly significant in the 40–90^°^C interval. This may correspond to the orderly changes in subcellular structural elements: proteins, ribosomes and RNA, membranes, and various structural elements of the cell wall during different points of the growth cycle and growth conditions. Spectral changes in the 90–105^°^C region are less pronounced, implicating that the structural composition of DNA-Protein (DNP) complexes may change little.

## INTRODUCTION

Differential scanning calorimetry is a bioanalytical technique, which, by measuring the melting process of different structural components of cells, provides directly the conformation change and other information of complex systems of biomolecules without the need of labor-intensive extraction of these biomolecules. Because of the simplicity of the experimental technique, it has long been used for the characterization of structures and biochemistry of complex biological systems[1,4,5,6,7,8] and understanding of biochemical responses to endogeneous[2,3] and exogeneous[9,10] stimuli. However, behind the simplicity of the experimental aspect of the method is the demanding interpretation of the complex calorimetric curves. This includes identification of separate clear peaks of thermoabsorption responsible for the melting of different subcellular structures in investigated samples. For example, comparative studies of differential scanning calorimeter (DSC) thermograms of various bacterial strains demonstrate that the thermograms consist of several distinct peaks of thermoabsorption due to the melting of different structural components within a cell[6]. Only a further comparison of the thermal denaturation of DNA (purified from *E. coli*) and the reversible thermoabsorption peaks of intact bacteria allowed one to resolve the peaks relative to the DNP complex. Mackey et al.[7] succeeded in demonstrating the relation between the melting temperature of intracellular DNA and its G-C content only after performing an intensive DSC investigation of more than 50 different bacteria types. Not until after a thorough study of intact cells of *E. coli* and its DNA component could Mackey et al.[8] succeed in associating thermoabsorption peaks with different thermal denatuation events of whole cell and different cell fractions.

In spite of the complexity associated with the interpretation of the complex calorimetric curves, Niven at al.[9] and Obuchi et al.[10] recently demonstrated that with clever designs of experimental conditions, DSC can potentially be a powerful tool to investigate mechanisms of cell survival when cells are exposed to environmental stresses. The work presented in this paper is the first of a series of calorimetric studies performed on bacterium *A. oxydans.* Our ultimate purpose is to identify cellular characteristics to its ability to survive in environments heavily polluted with the toxic hexavalent chromium Cr(VI) by reducing Cr(VI) to Cr(III) forms. The objective of this part of our research program was to develop a more efficient system for the differential scanning calorimetry of whole cells and their survival in hostile environments. Towards this end, we introduced a new concept of thermal spectrum. The DSC thermograms of complex biological systems traditionally consist of peaks associated with endothermic and exothermic processes. It is known that endothermic processes associate with intramolecular melting (and thus structure and conformation) of subcellular structures within a cell, whereas exothermic processes generally are associated with macromolecular synthesis *de novo* or with polymerization of certain subcellular structures. In our study, we selected measurement conditions (buffer system, scanning rate) so that exotherms were not observed. The thermograms, also called thermal spectrum in this paper, obtained in this manner are thermal curves, which depict only the endothermic events. Their spectral characteristics represent the temperature dependence of the increase in sample heat capacity as a result of intramolecular melting of individual biological active substances (proteins, nucleic acids, ribosomes, membrane, and other membrane structures). That enables us to separate absorption maximum related to DNP complex melting by graphical analysis of the whole thermal spectra. This paper describes the application of this new concept to characterize cell compositions of bacterium *A. oxydans* at their transition from logarithmic to stationary growth stage.

## METHODS

All DSC measurement conditions (buffer system, scanning rate) were selected so that only endotherms were observed. All thermal spectra were thermograms measured in the temperature range of 40–105°C, and were normalized by dry weight of sample after drying at 105 ± 2°C.

### Cell Culture Conditions

*A. oxydans* bacteria were isolated from basalt samples of Columbia rocks (U.S.) and grown under two culture systems. The first system involved growing *A. oxydans* in the standard medium recommended for *Arthrobacter* species[11] at the temperature of 21°C under constant shaking. Their growth was monitored by measuring the turbidity of the cell suspension at 440 and 590 nm. The second system involved growing cells on agar plates (10% agar prepared with the above mentioned medium). Spheroplasts of *A. oxydans* were obtained from colonies on plates according to the method[12].

### Determination of the Total Bacterial Protein

The protein concentration in total cell extract was determined by BCA Protein Assay (Pierce).

### Fluorescence Microscopy

Intact cells were stained with acridine orange (AO) (1:1)[13]. The staining was performed on unfixed cells on microscopic slides directly at room temperature for 5 min and then rapidly dried under a stream of warm air[14]. The complex morphological characteristics of *A. oxydans* and changes of cell metabolism at their transition from logarithmic to stationary phase were observed using a fluorescence microscope (Lumam – I2, LOMO, Russia) with an oil immersion objective.

### Sample Preparation for Calorimetry

Cell suspension was centrifuged at 10,000 rpm for 10 min. Pellet was washed several times in low ionic buffer solution at pH (7-7.5) prepared from 50 mM Tris, 0.1 M NaCl*,* 0.1 mM PMSF, 0.1 mM Benzamidine. The pellet was subjected to subsequent centrifugation. The pH range was selected because it did not influence the pattern of the thermal spectra. Afterwards, 0.2 ml of the pellet was placed into the stainless steel measurement cell. In the case of cell culture being grown on agar plates, cells were washed by rinsing the agar surface with the similar buffer solution, centrifuged, and then prepared in the same manner. Some cell suspension was also treated with high ionic buffer solution (0.55 M NaCl) for comparison. The consumption of the matter in the experiment was 5–15 mg of dry weight.

### Calorimeter

Calorimetric study was carried out by means of a modified device originally elaborated and designed in the Physics Institute of the Academy Science of Georgia[15]. It worked on line with a Pentium 2 computer. The maximum sampling frequency was 4 points/s. In this study, our measurements were carried out at a sampling frequency of 1 point per 2 s, at a heating rate of 35 K/h that gives a temperature resolution of 0.02 K. The accuracy of absolute temperature measurement is not less than 0.05 K over the temperature range of 5–150°C. The calorimeter resolution ratio in accordance with thermal capacity change (i.e., significant deviation from the basic line) is $10^{-5}$ J/K. With a time constant of 1 min, this enables one to resolve peak distance at no less than 0.6 K.

## Data Processing

The initial data processing (e.g., baseline approximation, calibration and curve normalization) was conducted using our in-house software. The final analysis (including identification of separate maximums, integration of curves and chart plotting) was conducted using program package Origin 6.0 (Microcal™ Software, Inc.).

## RESULTS AND DISCUSSIONS

All thermoabsorption measurements were conducted at the same point of growth stage of cell culture. All thermal spectra were reproducible for the whole temperature range of 40–105°C with a precision in the melting temperature no less than 0.1 K.

Fig. 1 shows a typical thermal spectrum (normalized by the sample dry weight) of bacteria *A. oxydans* from agar plates. Roughly it may be divided into two temperature intervals of 40–90°C and 90–105°C. The question arises what temperature interval corresponds to the melting of certain subcellular structures.

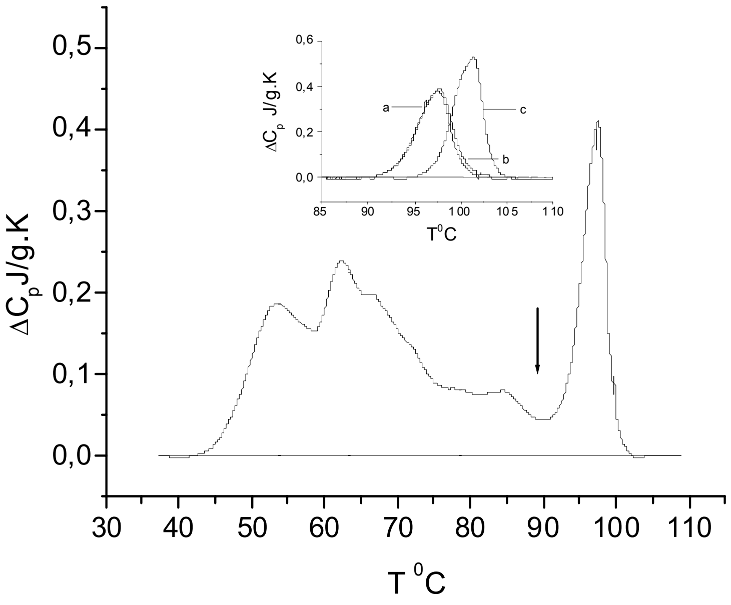

**FIGURE 1.** The thermal spectrum of bacteria *A. oxydans* grown on agar and measured on the fifth day of culture growth. The arrow points out the temperature to which the sample was heated. The insert shows the thermal spectra range reflecting DNP melting process in cells. Insert: curve a — experimental curve (0.1 M NaCl), b — curve singled out from the summary thermal spectrum with graphic analysis (0.1 M NaCl), c — curve singled out from the summary spectrum with graphic analysis (0.55 M NaCl).

From the recorded thermal absorption maximum (at 97.7°C) and the curve shape, it is most reasonable to suppose that cell DNP complex of *A. oxydans* melts in the temperature range of 90–105°C. Since only the structural elements that are in significant amount within a cell participate in the thermal spectrum formation, we can speculate that the rest of the cell structures (proteins, ribosomes and RNA, membranes, cell wall structural elements) melt in the range of 40–90°C. A process measured in the present experimental conditions was practically irreversible. If we heat the previous sample up to 90°C and then cool back to the room temperature, we get all cell compounds melted but DNP complex. After final heating up to 105°C, we will obtain the maximum related specifically to DNP complex melting, which is presented in the insert in Fig. 1. Curve (a) (the same insert) was obtained by processing the whole thermal spectra with graphic technique. Comparison of these two figures shows that our procedure for handling samples and analysis is perfectly reasonable. For further clarification of the nature of the cell structures being melted at high temperature interval, we also obtained the bacterial cell thermal spectrum after their brief exposure to buffer solution of high ionic strength (0.55 M NaCl) (curve c).

Thermodynamic parameters of the processes for mean thermograms of all three conditions are presented in Table 1.

**TABLE 1**
**Thermodynamic Parameters of the DNP Complex Melting Processes in Intact Cells Calculated for the Curves Presented on the Insert of Fig. 1**

| Curves | Q (J/g) | $T_m$ (°C) | ΔT (K) |
|---|---|---|---|
| a: Experimental (0.1 M NaCl) | 1.7 ± 0.1* | 97.7 | 4.1 |
| b: Graphic processed (0.1 M NaCl) | 1.8 ± 0.1 | 97.7 | 4.2 |
| c: Graphic processed (0.55 M NaCl) | 2 ± 0.1 | 101.3 | 3.6 |

**Total error was calculated as the error of dry weight estimation and the error of graphic processing.*

The shift of the thermoabsorption peak and the reduction of its width confirm our suggestion that the high temperature interval of the thermal spectrum reflects melting of the DNP complex of the investigated bacterial cell culture[16,17]. Heat of melting (see Table 1) corresponds well enough to those obtained for different bacteria presented earlier[6]. Interestingly the values of DNP melting heat obtained in this study are one degree less then that for DNA and chromatin for human blood cells[4]. These differences could be caused by the different ways of normalizing the thermal spectra. In this study, as it also had been performed for other bacterial strains[6], we normalized the spectrum by the total dry mass of sample (instead of by the dry mass of DNP complex).

Further analysis of thermal spectra for cells grown in different conditions and at different points of their growth cycle indicate that the DNP complex always melted at the same temperature interval under the same measurement conditions. The melting temperature and peak absorption intensity changed only very slightly.

Fig. 2 shows thermal spectra of bacteria from different points of their growth stage. Multiple measurements demonstrate that the spectral profile of this interval appear to be dependent on cell cultivation conditions and growth stage. This is a contrast to the intensity of DNP complex melting observed in the 90–105°C range that may be due to the thermal spectrum norming issue. This will be discussed below.

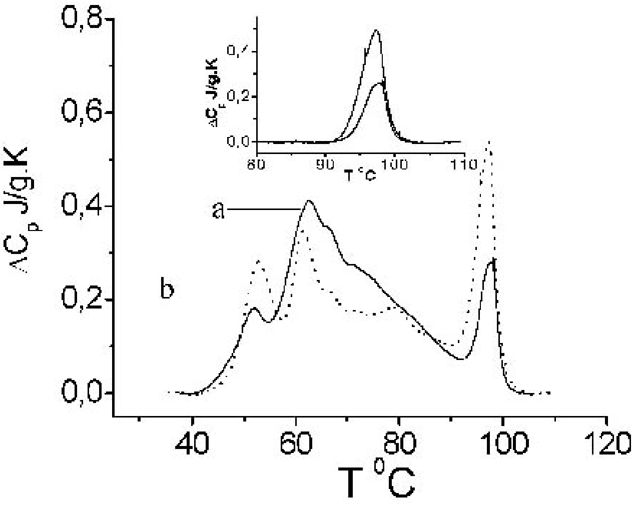

**FIGURE 2.** Thermal spectra of *A. oxydans* cells from different growth stages. (a) At the middle of the logarithmic phase; (b) at the beginning of the stationary phase. The insert shows spectra in the range corresponding to the DNP melting process (for comparison).

In Fig. 2, significant changes of the spectral characteristics are shown also in the temperature range of 40–90°C for bacterial cells in the middle of their logarithmic phase (spectrum a) and at the start of stationary phase (spectrum b). These changes reflect qualitative (and possibly quantitative) alterations of subcellular structures, which melt in this temperature interval. It is known[18] that cell wall composition is the most changeable at the development of the growth process. Thus one could suggest spectral changes in a certain part of this thermal spectrum interval arise from the melting of such cell structures. This speculation is supported by the thermal spectra of whole bacteria (Fig. 3a) and the spheroplasts (Fig. 3b). A comparative analysis of these spectra shows that the main part of the spectrum in the 40–90°C interval is due to the melting of structural elements of the cell wall, at the same time the thermal characteristics of DNP complex practically do not change. The melting of cytoplasmic structures can be identified at lower part within the 40–90°C region of the thermal spectrum b (Fig. 3).

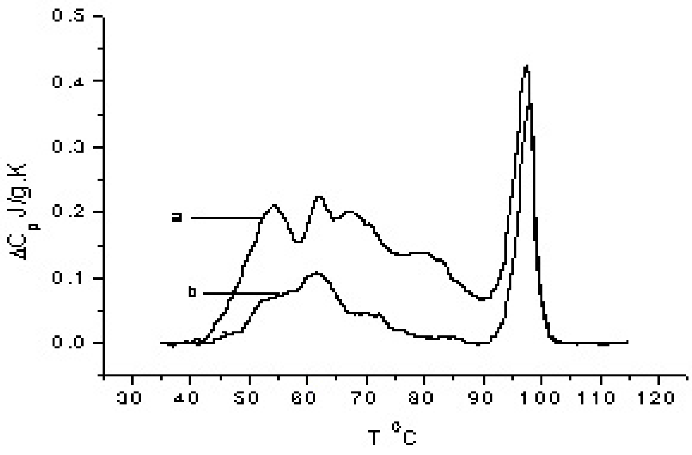

**FIGURE 3.** The thermal spectra of (a) whole cells of *A. oxydans*, (b) spheroplasts.

Similar to our earlier observations, the DNP melting process differs only slightly with growth of bacterial culture. The changes in the measured heat of the melting process might be related to the relative alteration of the DNA amount in the cell. As shown in Table 2, the measured heat of melting of whole cells ($Q_\Sigma$) and DNP complex ($Q_{DNA}$) and ratio $Q_{DNA}/Q_\Sigma$ demonstrate that the relative amount of DNA in *A. oxydans* at the stationary stage doubled compared to that at the logarithmic stage.

**TABLE 2**
**Melting Heat of Whole Intact Bacteria and DNP Complex of Thermal Spectra Presented in Fig. 2**

| Heat Spectra in Fig. 2 | Total Heat of Bacteria "Melting" $Q_\Sigma$ J/g | Heat of DNP "Melting" $Q_{DNA}$ J/g | $Q_{DNA}/Q_\Sigma$ | DNP "Melting" Temperature $T_m$ °C |
|---|---|---|---|---|
| a | 11.2 ± 1.1 | 1.0 ± 0.1 | 0.09 | 97.6 |
| b | 10.8 ± 1.0 | 2.0 ± 0.1 | 0.2 | 97.3 |

This observation may be explained by the relative increase of DNA in cells and/or decrease of the rest structural elements melting in the 40–90°C range. It is known that only one copy of DNA exists in the stationary phase[19]. It may be only more than one and never less than one DNA copy in logarithmic phase during cell division. Thus the spectra in Fig. 2 may not represent this biological process adequately. All thermal spectra should be normalized in such a way that the intensity of the thermoabsorption peak maxima (or, more correctly the areas under them) reflects DNP melting processes in intact cells, presuming that the DNA amount in cells does not change during culture growth.

The results of such alternative analysis are shown in Fig. 4. This was accomplished by normalizing all thermal spectra in Fig. 2a so that the thermoabsorption peak maximum due to the melting of the DNP complex of cells from different growth stages coincide with each other (see Fig. 4 a,b).

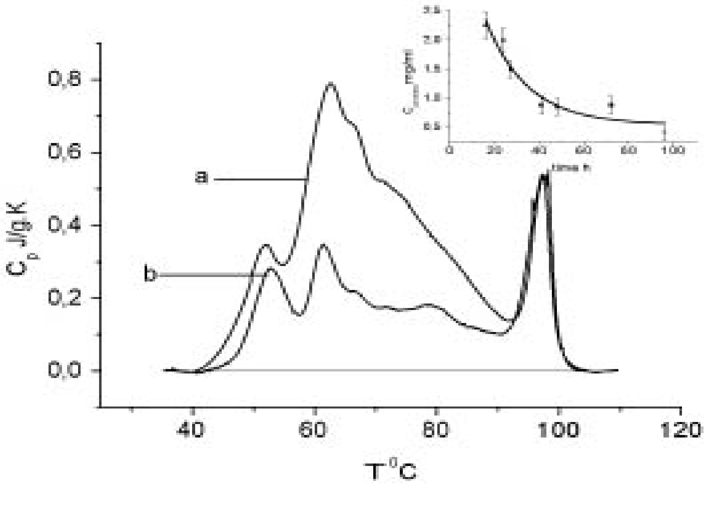

**FIGURE 4.** Thermal spectra of bacteria at different culture growth phases. (a) Middle of the logarithmic phase, (b) beginning of the stationary phase. Spectra are normalized by the amount of DNA. The insert shows total protein amount dependence on culture growth time.

As a result, the area under each thermal spectra in the 40–90°C range significantly increases. This means that the amount of cell structures that melts in this temperature range decreases as the culture undergoes the transition from logarithmic to stationary growth stage. This corresponds to the dependence illustrated by the insert in Fig. 4, reflecting the change of total protein amount as cells were growing. Similar results are presented by Strashinskaja et al.[20], wherein content of cell wall components decreased at the beginning of the stationary growth stage.

Microphotographs in Fig. 5 demonstrate evidence that support this interpretation. These microphotographs compare the relative content of cell nucleic acids at different growth stages of bacterial culture stained with AO. AO complexes with single- and double-stranded nucleic acid emit red (640 nm) and green (530 nm) fluorescence, respectively. It also has been shown to stain RNA in intact ribosomes[14]. Thus it provides the basis for simultaneous staining of RNA and DNA. The greater proportion of red fluorescing cells over green fluorescing cells on the presented photographs (Fig. 5a) indicates a high content of RNA. Green-fluorescing cells (Fig. 5b) are a predominantly stationary cell culture that is characterized by small number of ribosomes per cell.

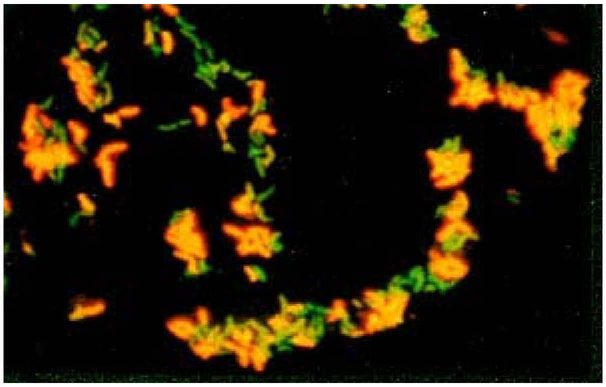

**a**

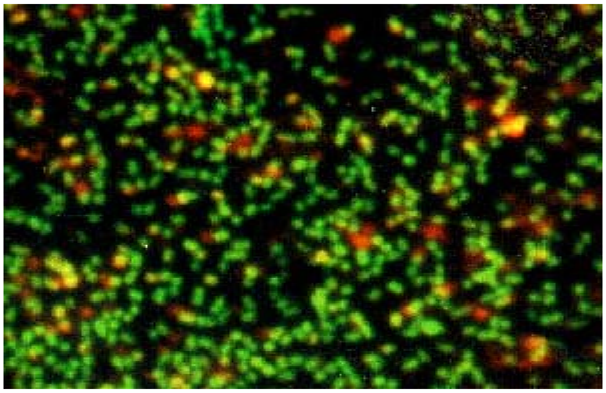

**b**

**FIGURE 5.** Microphotograph of bacteria: (a) at the middle of the logarithmic phase, (b) at the stationary phase (72 h of culture growth).

Further comparison shows the decrease of cell structures that “melt” in the 40–90°C interval as cell cultures were in transition from logarithmic to stationary growth stage. At the same time the DNP complex thermostability changes only slightly at growth development (see Fig. 4). The temperature shift is small (just 0.3 K), when compared with the insert of the Fig. 2 where the thermal spectra of DNP structure melting are shown, and with the correspondent temperatures of heat absorption maximum in Table 2. This shift is not a measurement error as was mentioned earlier; the reproducibility of cell DNP complex melting temperature was better than 0.1 K.

Cell transition from one phase to another — an adaptation process providing quick and reversible phenotype changing — is regulated on genome level[21]. Proteins, which take part in bacterial DNP structures and function, undergo quantitative and qualitative changes during cell transition from logarithmic to stationary phase[22]. In the case of *E. coli* W3110[22] it was shown that superhelical density of chromosomal DNA decreases in the late stationary stage of bacterial growth[23]. The decrease of the melting temperature of the *A. oxydans* DNP complex during transition from logarithmic to stationary stage may be really the reflection of such type of structural changes.

Our future study will use the new concept of thermal spectrum and the method of analysis developed in this paper to investigate the *survival mechanisms of metal-reducing bacteria* in heavily polluted environments.

## ACKNOWLEDGMENTS

This study was supported by ISTC grant N Ge-348.

## BIOSKETCHES

**Nugzar G. Bakradze,** Dr. Sci., is head of the Biological Physics Department, Andronikashvili Institute of Physics, AS of Georgia. He received a Ph.D. in biophysics from the Tbilisi State University, Georgia, USSR (1975) and Dr. Sci. in biophysics from the Institute of Biophysics, AS USSR, Pushchino, Moscow region (1987). His research efforts focus on the investigation of biopolymers and multiplex biological systems (including cells and tissues), physical behaviors using the methods of microcalorimetry, and NMR. He is the author of more than 80 articles for journals and conference proceedings.

**Marina K. Abuladze**, Ph.D., was a predoctoral fellow in the Laboratory of Cytogenecity and Cell Morphology at the Institute of Molecular Biology (Moscow). She received her doctorate in cytology from the Biological Department of Moscow State University in 1976. Currently, she is a Senior Researcher at the Andronikashvili Institute of Physics. Her work is centered on cell biology, particularly on cell proliferation and growth regulation. She is the author of more than 20 articles for biochemical and cell research journals.

**Victor M. Sokhadze**, Ph.D., is a scientist in the Biological Physics Department, Andronikashvili Institute of Physics, AS of Georgia. He received a Ph.D. in biophysics from the Andronikashvili Institute of Physics, AS of Georgia (1987). His work is dedicated to the investigation of the interaction between water and biomacromolecules by low temperature calorimetry method. He is the author of more than 40 articles for journals and conference proceedings.

**Nina V. Asatiani**, Ph.D., was a predoctoral fellow in the Laboratory of Protein Research at the Institute of Molecular Biology (Moscow). She received her doctorate in biochemistry from the Institute of Plant Biochemistry (Tbilisi, Georgia) in 1985. Currently, she is a Senior Researcher at the Andronikashvili Institute of Physics. Her research efforts focus on the biochemistry of chromatin, specifically the histone modifications. She is awarded by grants from ISTC. She is the author of more than 20 articles for biochemical, biophysical, and molecular biology journals.

**Nelly A. Sapojnikova**, Ph.D., was a predoctoral fellow in the Laboratory of Chromatin Research at the Institute of Cytology (St. Petersburg). She received her doctorate in biophysics from the Andronikashvili Institute of Physics (Tbilisi, Georgia) in 1989. Currently, she is a Senior Researcher at the Andronikashvili Institute of Physics. Her research efforts focus on the molecular biology of chromatin, specifically the active chromatin: (1) reduced levels and altered binding of linker histone group H1; (2) acetylation of core histones; (3) the presence of HMG1 protein. She was awarded by INTAS grant (1995–1996), by Wellcome Trust grant (1998-2001), by the grant from the Austrian Academy of Science. Currently, she is awarded by grants from ISTC. She is the author of more than 15 articles for biochemical, biophysical, and molecular biology journals.

**Tamar M. Kartvelishvili**, Ph.D., is a Senior Researcher in the Department of Peptide Synthesis at the Institute of Molecular Biology and Biophysics (Tbilisi, Georgia). Dr. Kartvelishvili received a B.S. (with honors in organic chemistry) in 1974 from the Tbilisi State University and Ph.D. in polymer chemistry from the Institute of Organoelement Compounds (INEOS, Moscow) in 1986. Her research interests focus on synthesis and study of new heterochain biodegradable polymers composed of naturally occurring α-aminoacids and other nontoxic building blocks, and containing enzymatically and chemically hydrolyzable ester links; synthesis of biodegradable functional elastomers for biomedical applications; investigation of mechanisms of intracellular chromium reduction and investigation of the cell metabolism pathways in the response of heavy metal actions. Dr. Kartvelishvili is the author of 38 articles.

**Emma N. Namchevadze** Ph.D., is a scientist of Biological Physics Department Andronikashvili Institute of Physics AS of Georgia Ph.D in biophysics from the Andronikashvili Institute of Physics AS of Georgia (1989). Her work in general was dedicated to investigation of interaction between water and biomacromolecules by NMR method. Now she is working on fluorescence microscope. She is the author of more then 15 articles for journals and conferences proceedings.

**Nelly Tsibakhashvili**, Ph.D., is a Senior Researcher at the Andronikashvili Institute of Physics. She received her Ph.D. in physics in 1990. Her research interests are focused on the study of environmental and biological samples using separation methods (capillary electrophoresis, gas chromatography). In 1993, she was awarded by Soros Grant. Since 1999 she is a member of the British Electrophoretic Society. At present she is ISTC Project Manager (G-348) - Study of molecular mechanisms of heavy metal transformation on microbial-mineral surfaces. N. Tsibakhashvili is the senior author of 32 original scientific publications.

**Leila V. Tabatadze** received a B.S. from Tbilisi State University in physics in 1975. Currently, she is a science researcher at the Andrinikashvili Institute of Physics.

**Lia V. Lejava** received a B.S. from Tbilisi State University in physics in 1975. Before starting graduate work, she served as the laboratory assistant at the Andronikashvili Institute of Physics. Currently, she is a science researcher in the same Institute.

**Hoi-Ying N. Holman** is a Principal Investigator at the Lawrence Berkeley National Laboratory, Berkeley, California. She received a Ph.D. in the School of Engineering, University of California at Berkeley.
